# FLAVIN-CONTAINING MONOOXYGENASES (FMOS): LONGEVITY-PROMOTING ENZYMES OR ATHEROSCLEROSIS RISK FACTOR?

**DOI:** 10.1093/geroni/igz038.3497

**Published:** 2019-11-08

**Authors:** Ryan J Rossner, Matt Kaeberlein

**Affiliations:** University of Washington, Seattle, Washington, United States

## Abstract

Since their discovery in 1970, flavin-containing monooxygenases (FMOs) have been studied as Phase 1 xenobiotic metabolizing enzymes that act on sulfur- and nitrogen-containing small molecules. In 2015, we demonstrated that C. elegans (worm) fmo-2 was not only necessary for hypoxic response and dietary restriction-mediated lifespan extension, but was also sufficient to extend lifespan when overexpressed. Consistenet with a conserved role for FMOs as longevity-promoting enzymes, mouse hepatic Fmo3 transcript is highly upregulated by numerous major lifespan extending interventions. A contrasting series of reports, however, have described mammalian Fmo3-mediated production of trimethylamine N-oxide (TMAO) as a risk factor for atherosclerosis and other major diseases. My thesis research aims to define the regulation and function of worm fmo-2 using genetic and biochemical approaches. My data thus far support the hypothesis that fmo-2 acts on sulfur amino acid pathway intermediates to promote longevity and healthspan.

